# Investigating the potential roles of intra-colonial genetic variability in *Pocillopora* corals using genomics

**DOI:** 10.1038/s41598-024-57136-5

**Published:** 2024-03-18

**Authors:** Nicolas Oury, Hélène Magalon

**Affiliations:** 1 UMR ENTROPIE (Université de La Réunion, IRD, IFREMER, Université de Nouvelle-Calédonie, CNRS), Université de La Réunion, 97744 St Denis Cedex 09, La Réunion France; 2 Laboratoire Cogitamus, Paris, France; 3 Laboratoire d’Excellence CORAIL, Perpignan, France; 4 Present Address: KAUST Red Sea Research Center and Marine Science Program, Biological and Environmental Science and Engineering Division, King Abdullah University of Science and Technology (KAUST), 23955-6900 Thuwal, Saudi Arabia

**Keywords:** Biodiversity, Evolutionary ecology, Molecular ecology, Evolutionary genetics, Evolutionary biology, Genomics, Mutation

## Abstract

Intra-colonial genetic variability (IGV), the presence of more than one genotype in a single colony, has been increasingly studied in scleractinians, revealing its high prevalence. Several studies hypothesised that IGV brings benefits, but few have investigated its roles from a genetic perspective. Here, using genomic data (SNPs), we investigated these potential benefits in populations of the coral *Pocillopora acuta* from Reunion Island (southwestern Indian Ocean). As the detection of IGV depends on sequencing and bioinformatics errors, we first explored the impact of the bioinformatics pipeline on its detection. Then, SNPs and genes variable within colonies were characterised. While most of the tested bioinformatics parameters did not significantly impact the detection of IGV, filtering on genotype depth of coverage strongly improved its detection by reducing genotyping errors. Mosaicism and chimerism, the two processes leading to IGV (the first through somatic mutations, the second through fusion of distinct organisms), were found in 7% and 12% of the colonies, respectively. Both processes led to several intra-colonial allelic differences, but most were non-coding or silent. However, 7% of the differences were non-silent and found in genes involved in a high diversity of biological processes, some of which were directly linked to responses to environmental stresses. IGV, therefore, appears as a source of genetic diversity and genetic plasticity, increasing the adaptive potential of colonies. Such benefits undoubtedly play an important role in the maintenance and the evolution of scleractinian populations and appear crucial for the future of coral reefs in the context of ongoing global changes.

## Introduction

Intra-colonial genetic variability (IGV) means the presence of more than one genotype in a single colony^1–3^, a condition challenging the definition of the colony as a single homogeneous genetic entity. These genotypes usually result from intra-organismal genetic modifications such as somatic mutations, mitotic recombination, mitotic gene conversion^4,5^, or gene duplications^6^, leading to the formation of a mosaic. However, they can sometimes come from the fusion or exchange of genetically distinct parts from different organisms^1^, usually in early development stages^1,7–9^, producing a chimera.

Mosaic genotypes usually differ by few alleles, while chimeric ones differ by more, but distinguishing both mechanisms genetically remains tricky and relies on good knowledge of the studied organisms (e.g. mutation and recombination rates, age, and other life history traits). Even when organisms are well-known, reverse mutations or fusions between closely related individuals challenge the detection of mosaics. Several approaches involving more or less arbitrary defined genetic thresholds^10,11^ or based on Bayesian clustering^3^ have thus been proposed to genetically detect IGV, reporting a high prevalence in colonial taxa such as tunicates^12–15^, bryozoans^16^, sponges^17^, hydrozoans^18–21^, alcyonaceans^8^ or scleractinians^3,11,22–26^.

IGV in scleractinians has been increasingly studied over the past decade, resulting both from its recent discovery in those organisms and from the need to address the alarming coral decline^27^. Several studies hypothesised that IGV could be a lifeline for corals, increasing their adaptive potential^28^. Indeed, IGV has long been seen as detrimental due to antagonistic interactions among the different genotypes (as for tumours and autoimmune diseases^29–31^), but recent studies highlighted some promising benefits of having multiple genotypes (e.g., improved growth^32^ or competitive abilities^33^). Identifying and quantifying those benefits therefore appear necessary, but most of the previous studies only focused on quantifying the occurrence of IGV in natural populations without providing additional insights on its role.

In this study, we focused on *P.* *acuta* in the southwestern Indian Ocean (Reunion Island), a species that is abundantly found in shallow waters and is able to propagate asexually^34–36^. Although easily accessible, and thus intensively studied, knowledge about this species remains limited, partly due to past misidentifications. It has been widely confused with *P.* *damicornis*, of which it has recently been redefined^37^. However, its validity as a single species remains debated, and several studies^34,35,38,39^ delimited multiple genetic entities within it, suggesting that it could be a species complex. In the southwestern Indian Ocean, two secondary species hypotheses (SSH05c and SSH05d), were delimited based on 13 microsatellites^40^. Moreover, SSH05c showed a deeper partitioning into two diverging, but sympatric, genetic groups (Cluster 1 and Cluster 2, a posteriori named SSH05c-1 and SSH05c-2, respectively)^11,34,35^, whose existence has recently been confirmed using genomic data^38,39^.

IGV was first identified in *Pocillopora* corals using histocompatibility and allorecognition observations^41,42^. More recently, it was investigated using microsatellites in *P.* *damicornis *sensu lato (i.e. the species complex, before the latest taxonomic revision^37^) larvae from Thailand and Philippines^22^ and in *Pocillopora* spp. colonies (a mix of *P.* *damicornis *sensu stricto, *P.* *acuta*, and unidentified *Pocillopora* colonies) from Australia^3^. In Reunion Island, a first investigation using 13 microsatellites highlighted a high occurrence of IGV (up to 58%) in three *P.* *acuta* populations, mostly due to mosaicism (80% of IGV colonies)^11^. Such occurrence suggests potential, positively selected, benefits. Here, in order to further study IGV in these populations and characterise its potential roles from a genetic point of view, we sequenced the same colonies as in Oury et al.^11^ using target-capture of ultraconserved elements (UCEs) and exon loci. The importance of the bioinformatics pipeline in the detection of IGV was first assessed. Then, intra-colonial allelic differences were identified and characterised to highlight the potential impacts of these differences on the involved genes.

## Methods

### Sampling

**Figure 1:**
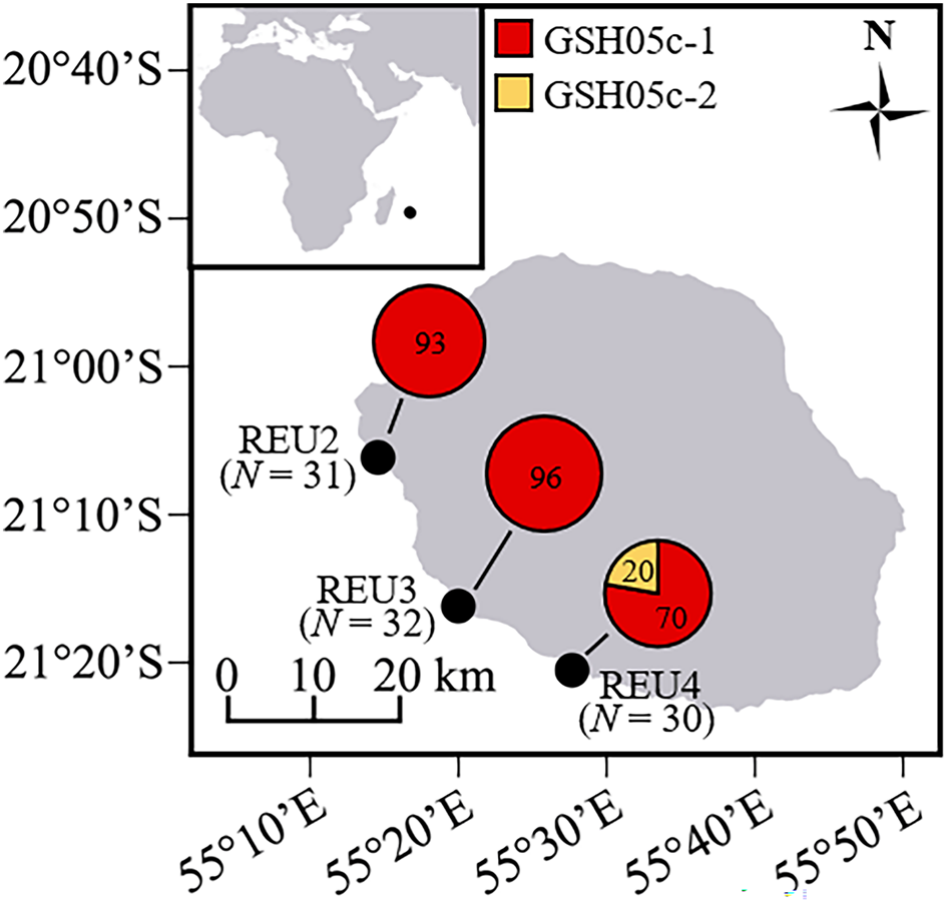
Sampling sites (black dots) of *Pocillopora acuta* colonies in Reunion Island (number of colonies in parentheses). For each site, the distribution of the number of nubbins (three per colony) per genomic species hypothesis (GSH sensu Oury et al.^38^) is indicated. Map generated in R v4.0.4^49^.

The samples used in the present study are exactly those that were previously studied to detect IGV in Oury et al.^11^ with microsatellites, except that we only included those genetically assigned to *Pocillopora acuta* (*N* = 94 colonies minus one; see below). Briefly, on each of three sites (REU2, REU3, and REU4 in Gélin et al.^34^) of the west coast of Reunion Island (southwestern Indian Ocean; Fig. 1), 32 adult (diameter > 10 cm) *Pocillopora* colonies were threefold-sampled. The three nubbins (< 1 cm^3^ each) within a colony were collected from apical branch tips by maximising the distance among them to enhance the probability of discovering multiple genotypes. Colonies were photographed and their surface area was approximated in situ by measuring the longest horizontal length possible on the upper side and the largest corresponding perpendicular width. Of the 96 colonies sampled, species identification was performed a posteriori using genetic assignment tests and 94 colonies were previously assigned to SSH05c (sensu Gélin et al.^40^ and corresponding to *P.* *acuta*)^11^. More precisely, 80 and 14 colonies were assigned to SSH05c-1 and SSH05c-2, respectively^11^. The two other colonies, in REU4, were assigned to *P.* *verrucosa* (SSH13a) and thus removed. Finally, 93 out of the 94 colonies assigned to SSH05c were considered in this study (one colony from REU2 was randomly removed to allow the inclusion of sequencing replicates in the sequencing library; Fig. 1).

### Laboratory and preliminary bioinformatics steps

Total genomic DNA of each nubbin was previously extracted using the DNeasy^®^ Blood & Tissue kit (QIAGEN GmbH, Hilden, Germany), following the manufacturer protocol. DNA quality and concentration were assessed using a NanoDrop™ 2000 spectrophotometer (Thermo Fisher Scientific, Wilmington, DE) and a Qubit^®^ 2.0 fluorometer (Invitrogen, Carlsbad, CA), respectively. Library preparation was then performed at the platform iGenSeq (ICM, Paris, France), following a capture protocol targeting 1248 ultraconserved elements (UCEs) and 1385 exon loci^43^, as in Oury et al.^38^. The resulting library was PE150 sequenced with an Illumina NovaSeq 6000 (Illumina, San Diego, CA). Three nubbins (one from one colony of each site) were independently prepared and sequenced three times each from the same DNA extract (sequencing replicates) to estimate the sequencing error rate. No extraction replicates were included as they would have been extracted from sub-samples of a single nubbin and can thus present IGV.

Following sequencing, reads were demultiplexed according to individual-specific indexes (no mismatch allowed), then quality checked with FastQC v0.11.7 (http://www.bioinformatics.babraham.ac.uk/projects/fastqc/) and MultiQC v1.7^44^, before and after adapter contamination and low-quality bases removal with cutadapt v2.1^45^, available in the wrapper script Trim Galore! v0.6.0 (http://www.bioinformatics.babraham.ac.uk/projects/trim_galore/; parameters are listed in Table S1).

### Species identification of the nubbins

Using genomics, recent studies investigating the limits of *Pocillopora* species^38^ and their population structure in the southwestern Indian Ocean^39^ highlighted a partitioning slightly different from the one previously identified with microsatellites for *P.* *acuta*. Thus, in order to confirm the species of the sampled nubbins in the light of these latest results, we first called the genotypes of the nubbins for the single-nucleotide polymorphisms (SNPs) used in these previous studies and performed assignment tests combining the genotypes included in these previous studies (available at 10.5281/zenodo.7885458 and 10.5061/dryad.pnvx0k6vw) and those from this study. The present genotypes were first assigned with those from Oury et al.^38^ to identify nubbins belonging to *P.* *acuta *sensu lato, and then with those from Oury et al.^39^ to distinguish the different genomic species hypotheses (GSHs) among the *P.* *acuta* species complex (i.e. GSH05c-1, GSH05c-2, and GSH05d in the southwestern Indian Ocean).

Briefly, trimmed reads were mapped to the 2068 reference sequences from Oury et al.^38^ using BWA v0.7.17^46^, following sorting and duplicates marking with Picard v2.20.7 (https://broadinstitute.github.io/picard/) and local realignment with The Genome Analysis Toolkit (GATK) v3.8.1^47^, as in Van der Auwera et al.^48^ (see Oury et al.^38^ for more details). Genotypes from the 1559 SNPs used for species delimitation^38^ and the 1493 SNPs used for exploring *P.* *acuta* population structure in the southwestern Indian Ocean^39^ were called with BCFtools v1.9 (http://samtools.github.io/bcftools/), requiring a minimum read depth (DP) of 12 × and non-significant strand biases (SP < 13). Three nubbins (from three distinct colonies) with lots of missing data (> 99%) were discarded (Tables S1, S2).

As many nubbins (both within and between colonies) were previously found to share the same multi-locus genotype (MLG) using microsatellites^11^, genomic clonal lineages were thus identified. Genetic distances among all pairs of nubbins were computed as the number of different alleles [estimated with the *diss.dist* function from the R v4.0.4^49^ library *‘poppr’*^50^] over the number of comparable sites (i.e. genotyped for both nubbins) for each pair. Distributions were then plotted to detect the antimode which corresponds to the threshold separating intra- from inter-clonal lineage comparisons, allowing to group nubbins within the different clonal lineages. Clonal lineages were then visualised with a hierarchical clustering of the individuals based on genetic distances. From that, only one representative of each clonal lineage was kept for assignment tests to avoid biasing clustering in favour of highly related individuals. The resulting truncated datasets (Table S2) were combined with those from previous studies^38,39^.

Assignment tests were performed with sNMF^51^, implemented in the R library *‘LEA’*^52^. Five repetitions per *K*, with *K* varying from 2 to 10, were run, with a maximum of 500 iterations before reaching stationarity, and results were visualised with CLUMPAK^53^. Nubbins from this study were identified according to the assignments (see Results).

### Intra-colonial genetic variability analysis

For further analyses, we used the genome of *P. verrucosa*^54^ as reference, as being the closest available annotated genome at the time of the analyses. Indeed, among the three available *Pocillopora* genomes, two were mislabelled as *P. acuta*^55^ and *P. damicornis*^56^, while corresponding rather to *P.* *verrucosa* and *P.* *grandis* (senior synonym of *P.* *eydouxi*), respectively^38^. Consequently, we chose the most assembled and annotated of the two genomes of *P.* *verrucosa*, this species being phylogenetically closer to *P.* *acuta* than *P. grandis*^38^. Trimmed reads were mapped to the genome as previously for the reference sequences (Table S1).

#### Intra-colonial genetic variability detection

To evaluate the impact of both variables, several SNP calling and filtering parameters were tested (Table 1) and the percentage of IGV colonies was represented as a function of the dissimilarity threshold. The pipeline retained for further analyses is presented below with tested parameter values indicated in Table 1.

**Table 1:** Single-nucleotide polymorphism (SNP) calling and filtering parameters tested for the detection of intra-colonial genetic variability (IGV).

Tested parameter	*N*_*SNP*_	T_50_	*Δrep*
Mapping & base qualities (MQ & BQ)			
MQ ≥ 10 & BQ ≥ 30	9 925 706	6.20%	7.31 ± 0.36%
MQ ≥ 20 & BQ ≥ 20	10 210 117	6.37%	7.49 ± 0.36%
**MQ ≥ 20 & BQ ≥ 30**	**9 895 232**	**6.11%**	**7.23 ± 0.36%**
MQ ≥ 30 & BQ ≥ 30	9 845 096	6.01%	7.12 ± 0.36%
Quality score (QUAL)			
& QUAL ≥ 5	8 684 717	6.24%	7.39 ± 0.37%
& QUAL ≥ 10	7 819 252	6.53%	7.66 ± 0.37%
**& QUAL ≥ 20**	**7 247 330**	**6.88%**	**8.07 ± 0.40%**
Depth of coverage (DP)			
& DP ≥ 6	161 872	1.55%	1.36 ± 0.07%
**& DP ≥ 12**	**60 062**	**0.95%**	**0.82 ± 0.04%**
& DP ≥ 20	43 187	0.70%	0.76 ± 0.05%
Strand bias (SP)			
**& SP ≤ 13**	**59 032**	**0.89%**	**0.76 ± 0.05%**
Site percentage of missing data (*%NA*)			
& *%NA* < 50%	34 220	0.74%	0.65 ± 0.03%
& *%NA* < 20%	27 974	0.57%	0.53 ± 0.02%
& *%NA* < 10%	24 893	0.46%	0.44 ± 0.01%
& *%NA* < 5%	22 165	0.36%	0.36 ± 0.01%
Minor allele frequency (MAF)			
& MAF ≥ 0.01	54 930	0.95%	0.82 ± 0.05%
& MAF ≥ 0.05	42 108	1.17%	0.98 ± 0.07%
& MAF ≥ 0.1	34 916	1.19%	0.98 ± 0.06%

Retained parameters are indicated in bold (see Results). *N*_*SNP*_: number of filtered SNPs, T_50_: dissimilarity threshold at which 50% of the colonies presented IGV, and *Δrep*: mean (± s.e.) dissimilarity between sequencing replicates of the same nubbin.

SNPs were called with BCFtools, treating all individual bam files simultaneously and requiring minimum base and mapping qualities (BQ and MQ, respectively) of 20 and 30, respectively. Sites were then filtered based on quality score (QUAL ≥ 20), while filtering based on minimum read depth (DP ≥ 12 ×) and strand bias (SP ≤ 13) was carried out at the genotype level. Tri- and tetra-allelic sites were also discarded as they were not supported in some later analyses. The three nubbins with lots of missing data were systematically discarded, regardless of the parameters tested. Filtering on site percentages of missing data and on minor allele frequencies (MAF) were also tested but not retained for further analyses (Tables 1 and S1). Each resulting VCF file was then analysed using a custom R script calculating the percentage of different alleles between all pairs of intra-colonial nubbins with the *diss.dist* function from the library *‘poppr’*^50^ (but taking into account the number of comparable sites, i.e. without missing data for both compared nubbins). The maximum distance between intra-colonial nubbins was then retained and used to consider whether the colony presented IGV in function of the dissimilarity threshold. To facilitate comparisons among parameters, the dissimilarity threshold at which 50% of the colonies presented IGV (T_50_) was calculated.

Finally, for the VCF resulting from the retained filtering steps (i.e. those described above; Table 1), an approach similar to the one used in Oury et al.^11^ was performed to help define the thresholds distinguishing (1) colonies presenting IGV or not and (2), among colonies presenting IGV, mosaic or chimeric colonies. All nubbins were compared by pair and the distribution of the percentage of differing alleles was plotted. Based on the same reasoning as the one used with microsatellites^11^, the distribution was expected to be trimodal: the first and second modes, in low values, should correspond to sequencing and bioinformatics errors, and to somatic mutations, respectively, while the third mode, in higher values, should correspond to chimerism. The threshold distinguishing colonies presenting IGV or not should therefore be the first antimode of the distribution and the one distinguishing mosaic from chimeric colonies, the second. However, using genomic data, the first and second modes are likely to overlap as genotyping errors may vary among samples. This can encrypt the first antimode corresponding to the threshold distinguishing colonies presenting IGV or not. Therefore, for low values, the distribution was decomposed into two Gaussian components (one corresponding to genotyping errors and the other to mutations) using a Gaussian mixture model (GMM) and the R library *‘mixtools’*^57^. The first antimode distinguishing colonies presenting IGV or not was defined at the intersection of both Gaussian density curves. Finally, once thresholds were identified, the proportions of invariable, mosaic, and chimeric colonies were calculated per sampling site and per GSH, and compared with Fisher exact tests in R. We also tested whether colony upper surface area, as a proxy of colony age and/or growth rate, but also of distance between nubbins, could facilitate IGV, by performing Pearson correlation tests between colony surface area (length × width) and mean percentage of different alleles among intra-colonial chimeric or mosaic nubbins.

#### Intra-colonial genetic variability characterisation

Once chimeras were identified, mosaic and chimeric intra-colonial pairs of nubbins were distinguished in order to describe both processes from a genetic point of view. SNPs with different allelic states within colonies were identified and characterised based on reference genome annotations^54^ using a custom R script. Noteworthy, since we were unable to confidently distinguish SNPs resulting of genotyping errors from SNPs reflecting true allelic differences, even after SNP filtering, all SNPs diverging within colonies were characterised. Briefly, SNP positions were matched to annotated gene and associated coding region positions to identify coding SNPs. When appropriate, corresponding reference codons were identified and compared to alternate codons to quantify silent, nonsense, and missense allelic differences.

Finally, gene ontology (GO) terms previously assigned^54^ and describing the biological processes of the genes impacted were reduced with REVIGO^58^ to highlight the main processes potentially influenced by mosaicism or chimerism. GO terms were weighted by the number of non-silent allelic differences affecting the corresponding genes. To ease interpretations and efficiently reduce GO annotations, only terms represented by at least 50 mutations were considered and a redundancy cut-off of 0.4 was set. Dendrograms based on the dissimilarity of the reduced GO terms were then reconstructed in R for visualisation purposes.

## Results

The NovaSeq platform produced a total of 1.6 × 10^9^ reads (2.4 × 10^11^ bp), with between 1.3 × 10^6^ and 9.8 × 10^6^ reads per sample [mean ± s.e. = (5.7 ± 0.1) × 10^6^ reads]. Quality controls and adapter trims then led to the overall removal of 2.9% of the bases (from 1.1 to 10.2% per sample, except for three samples for which > 75% of the bases were removed and which were discarded for further analyses).

### Species identification of the colonies

Between 55.9 and 92.2% [mean ± s.e. = 84.1 ± 0.4%] of the trimmed reads per sample were successfully mapped on the reference sequences, with a mean coverage depth (± s.e.) of 74.9 × (± 1.7). The genotyping of the 1559 SNPs^38^ and the 1493 SNPs^39^ led to two datasets of 282 nubbins (after removing the three with a lot of missing data) with 3.6% and 3.2% missing data, and mean SNP coverage depths (± s.e.) of 111.6 × (± 1.9) and 113.9 × (± 1.9), respectively (Table S2).

For both datasets, the dissimilarity between sequencing replicates did not exceed 0.6% (mean ± s.e. = 0.2 ± 0.0% and 0.4 ± 0.1% for the 1559 SNPs and 1493 SNPs, respectively; Fig. S1), and the distributions of the pairwise percentages of different alleles among nubbins showed clear antimodes (no comparison between ~ 1% and 5–8%; Fig. S1). Thus, considering a threshold of 1% to distinguish nubbins of the same clonal lineage from nubbins of different ones (i.e. nubbins differing from less than 1% belong to the same clonal lineage), all 282 nubbins fell in a total of 15 clonal lineages, represented by one to 93 nubbins (Fig. S2). Most interestingly, 11 colonies had nubbins belonging to different clonal lineages, indicating potential chimeras (six of them were already detected as chimeras^11^).

Assignment tests with the species delimitation dataset^38^ confirmed that all nubbins belong to *P.* *acuta* (GSH05 sensu lato; Fig. S3a). Then, from the assignment tests with the population structure dataset^39^, 11 (259 nubbins) and 4 (20 nubbins) clonal lineages were assigned (*P* > 0.75, except for one clonal lineage) to GSH05c-1 and GSH05c-2, respectively (Fig. 1 and S3b). Meanwhile, 86 and 6 colonies had all their three nubbins assigned to the same GSH (GSH05c-1 or GSH05c-2, respectively), while the remaining colony over the 93 sampled (identified as chimera C9 in Oury et al.^11^), had one nubbin assigned to GSH05c-1, and the two others to GSH05c-2.

### Intra-colonial genetic variability analysis

#### Intra-colonial genetic variability detection

About 99.1% of the individual reads were mapped to the *P.* *verrucosa* genome, from which biallelic SNPs were called and filtered with the different parameters tested, resulting in 22,165 SNPs to 10,210,117 SNPs per dataset (Table 1). Noteworthy, for each SNP dataset, 11 colonies (matching those previously detected with nubbins belonging to different clonal lineages) systematically showed a maximal distance between intra-colonial nubbins about 10 times higher than other colonies (Fig. S4), again indicating potential chimeras. To better visualise the effect of filtering parameters on the detection of IGV, and as these colonies are undoubtedly variable, the much distant nubbin from the two others was discarded for each colony to only keep comparisons between mosaic or non-genetically different nubbins.

**Figure 2:**
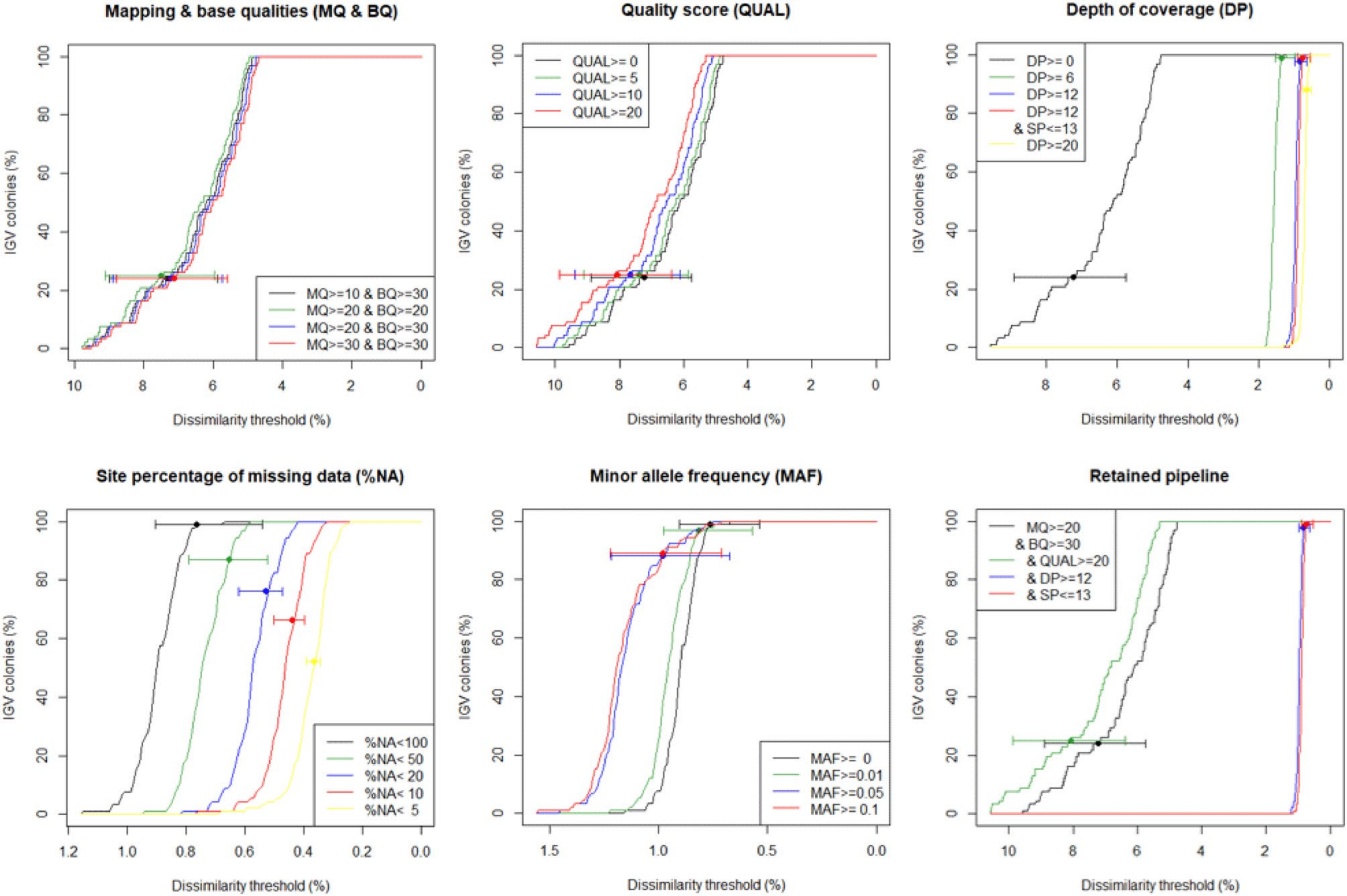
Effect of single-nucleotide polymorphism (SNP) calling and filtering parameters on the detection of intra-colonial genetic variability (IGV). Percentage of IGV colonies as a function of the threshold in percentage of different alleles between nubbins. Dots and associated whiskers indicate means (over nine comparisons) and ranges of pairwise distances among sequencing replicates of the same nubbins, respectively.

The different mapping and base quality values gave very similar results, as quality scores did. Percentages of IGV colonies varied in the same way as a function of the dissimilarity threshold, whatever the values tested, and T_50_ (i.e. the dissimilarity threshold at which 50% of the colonies present IGV) ranged from 6.01 to 6.88% (Table 1; Fig. 2). Conversely, filtering on depth of coverage (DP) at the genotype level had a great impact, starting from the least strict filter (DP ≥ 6). T_50_ varied from 6.88% for the dataset without filter on DP to 1.55% for the one with DP ≥ 6. More importantly, the curve changed from a stairway to a sigmoid and the mean (± s.e.) dissimilarity between sequencing replicates (*Δrep*) varied from 7.23 ± 0.36% to 1.36 ± 0.07%. Increasing the value of the DP filter did not significantly impact the detection of IGV (0.70% ≤ T_50_ ≤ 1.55%; 0.76 ± 0.05% ≤ *Δrep* ≤ 1.36 ± 0.07%), nor applying an additional filter on significant strand biases (SP ≤ 13; T_50_ = 0.89%; *Δrep* = 0.76 ± 0.05%; Table 1; Fig. 2). Finally, filtering on site percentages of missing data (*%NA*) and minor allele frequency (MAF) had a minor impact (0.36% ≤ T_50_ ≤ 0.89% and 0.89% ≤ T_50_ ≤ 1.19%, respectively), but progressively removing sites with lots of missing data decreases the mean (± s.e.) dissimilarity between sequencing replicates (*Δrep* = 0.65 ± 0.03% when no filter is applied; *Δrep* = 0.36 ± 0.01% with *%NA* < 5%; Table 1; Fig. 2). Further analyses were performed on the dataset resulting from the following filters: MQ ≥ 20, BQ ≥ 30, QUAL ≥ 20, DP ≥ 12, and SP ≤ 13. While many of these filters do not seem to impact the detection of IGV (except DP; Fig. 2), they allowed us to efficiently reduce genotyping errors while limiting the number of rejected SNPs (Table 1). The dataset for further analyses therefore comprises 276 nubbins (without replicates) and 59,032 SNPs with 40.9% missing data and a mean SNP coverage depth (± s.e.) of 46.3 × (± 0.2).

**Figure 3:**
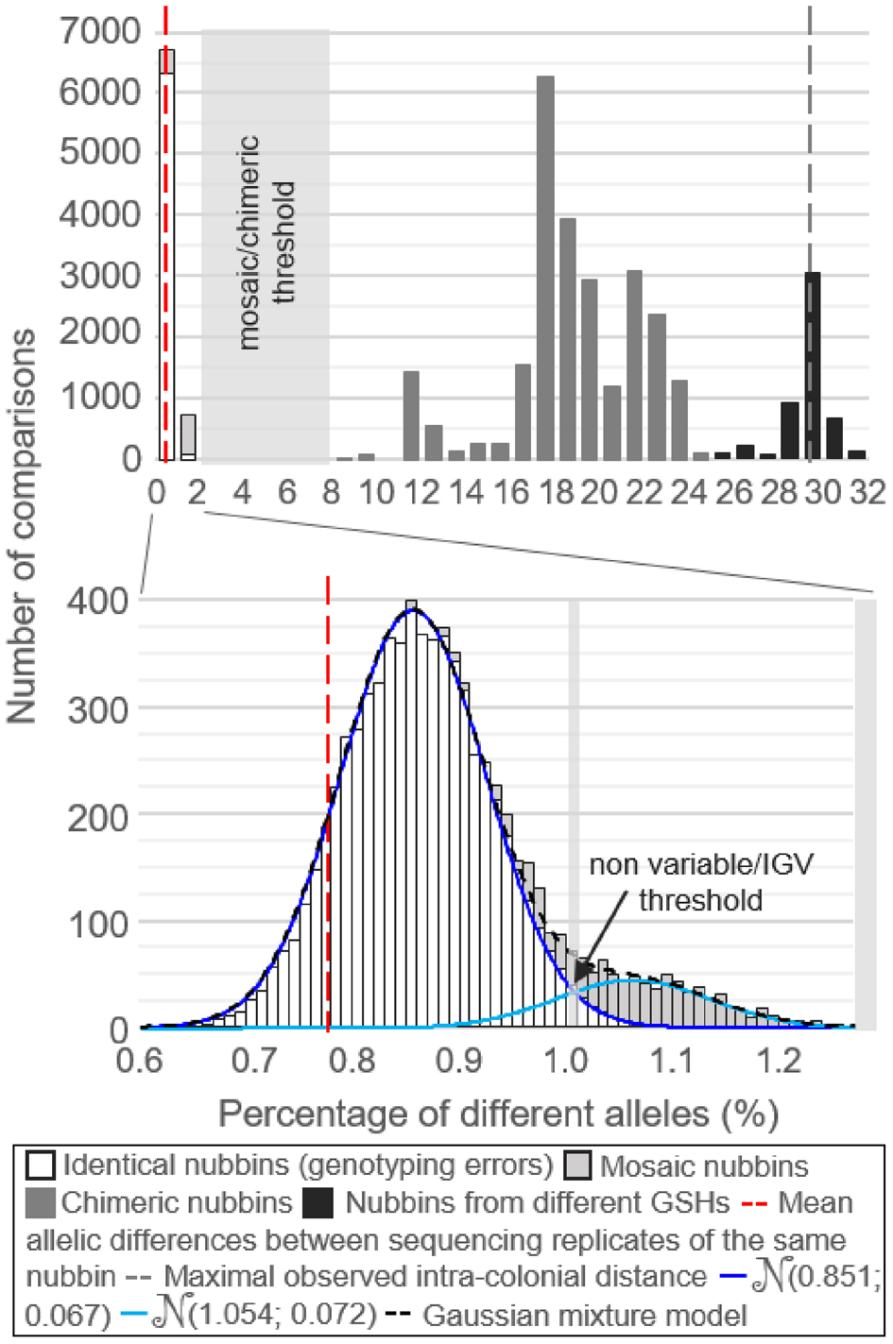
Distribution of the percentages of different alleles between all pairs of nubbins, with a zoom window between 0 and 2%. GSHs: genomic species hypotheses.

A total of 37,950 pairs of nubbins were compared, and the distribution of the percentage of differing alleles was plotted: at first glance, this distribution showed three modes and two antimodes, with no comparison found between 1.3 and 8.4%, nor between 24.4 and 25.2% (Fig. 3). However, the first antimode directly represented the threshold distinguishing mosaicism from chimerism, and the second separated intra-GSH (*N* = 32,830) from inter-GSH (*N* = 5120) comparisons (Fig. 3). Accordingly, the three modes corresponded to comparisons between (from lowest to highest percentages of differing alleles) identical and mosaic nubbins, chimeric nubbins, and nubbins from different GSHs. As expected, differences due to genotyping errors and mutations overlapped somewhere between 0.6 and 1.3%. The GMM decomposed the distribution in this range into two Gaussian components: (μ = 0.851%; σ = 0.067%) and (μ = 1.054%; σ = 0.072%), accounting for 89.0% and 11.0% of the nubbin pairs, respectively (Fig. 3). The corresponding Gaussian density curves intersected at 1.0%, which was defined as the threshold to distinguish colonies presenting IGV or not (i.e. below this threshold, differences were considered as sequencing errors; Fig. 3).

**Figure 4:**
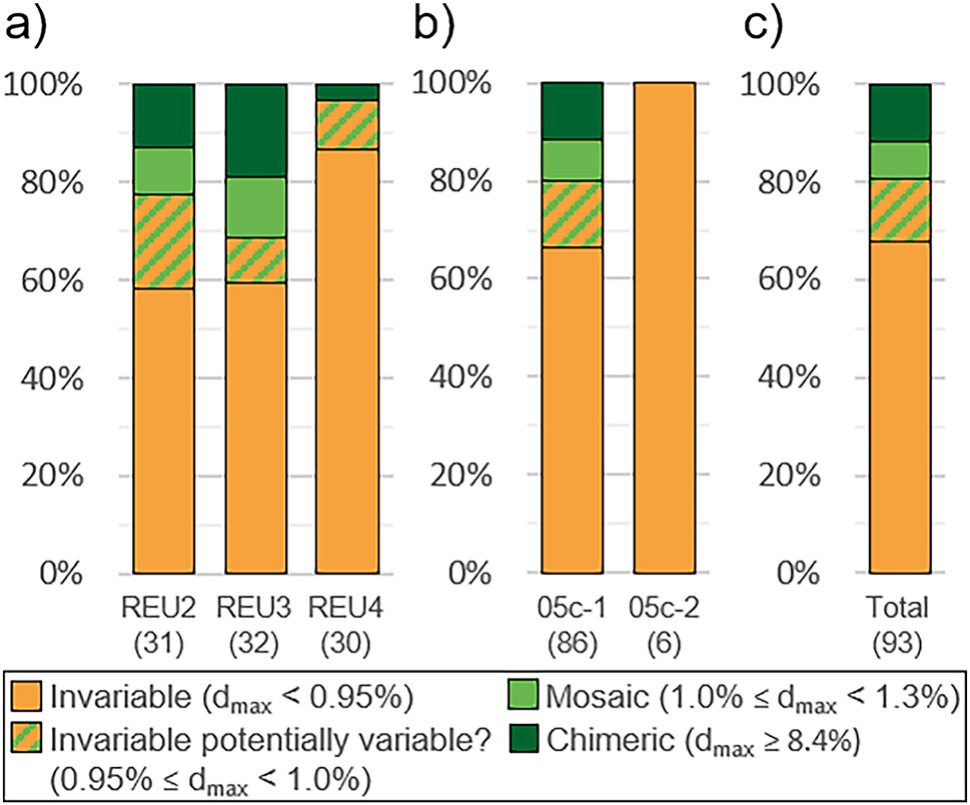
Proportions of the categories of genetic variability (**a**) per sampling site, (**b**) per genomic species hypothesis (GSH), and (**c**) overall colonies (number of colonies in parentheses). Distributions are not significantly different among sampling sites (Fisher exact test; *P* = 0.06) nor between GSHs (Fisher exact test; *P* = 1.00).

Considering this threshold of 1.0% to distinguish whether colonies present IGV, 18 colonies presented IGV (REU2: 7; REU3: 10; REU4: 1). According to the second threshold of 1.3–8.4%, 7 (REU2: 3; REU3: 4; REU4: 0) were mosaics and 11 (REU2: 4; REU3: 6; REU4: 1) were chimeras (Fig. 4). However, the numbers of IGV and mosaic colonies were extremely sensitive to the threshold defined, since a reduction of 0.05% of this threshold (i.e. 0.95% instead of 1.0%) increased these numbers by 66% (from 18 to 30 colonies) and 171% (from 7 to 19), respectively. Except the chimeric colony with nubbins assigned to both GSH05c-1 and GSH05c-2, all six remaining GSH05c-2 colonies were invariable, but no significant difference in the proportions of invariable, mosaic, and chimeric colonies was found between GSHs (Fisher exact test; *P* = 1.00), probably due to the unequal number of colonies belonging to each GSH. No significant difference was also found among sampling sites (Fisher exact test; *P* = 0.06).

Finally, no clear correlation was found between colony surface area and mean intra-colonial percentage of different alleles, for both chimerism (*N* = 11, *R*^2^ = 0.001, *P* = 0.904; Fig. S5a) and mosaicism (*N* = 92, *R*^2^ = 0.064, *P* = 0.016*; Fig. S5b), although it was significant for the latter due to the relatively high number of observations.

#### Intra-colonial genetic variability characterisation

Considering the 11 chimeras, 21 pairs of intra-colonial chimeric nubbins were identified. Indeed, each colony had two pairs of chimeric nubbins, consisting of the most distant nubbin with one of the two others (these latter being potentially mosaic between themselves). However, one nubbin was removed for a chimera due to missing data, so only one pair was identified. Conversely, 252 pairs of intra-colonial nubbins that were potentially mosaic were identified [242 for the 82 non-chimeric colonies (two had only two nubbins) and the 10 pairs resulting from the less distant nubbins within chimeras].

**Figure 5:**
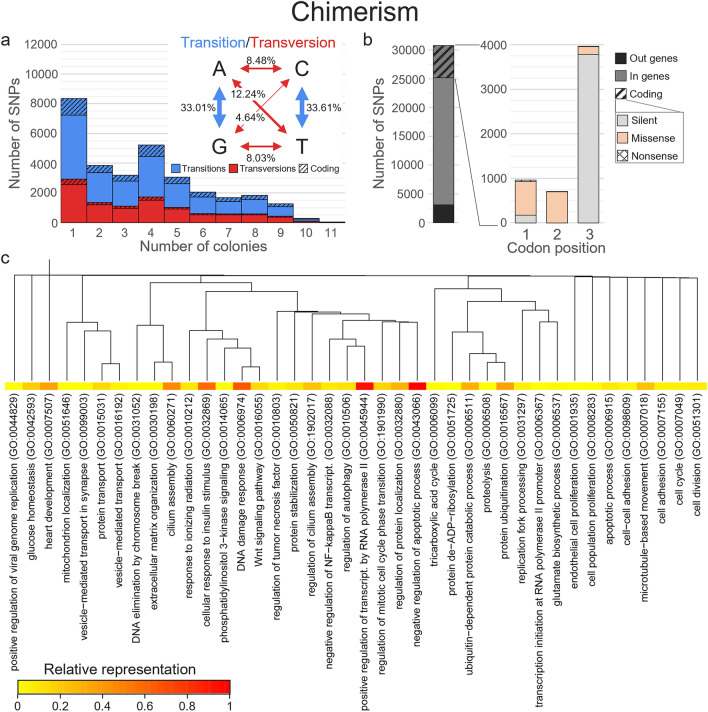
Characterisation of chimerism. (**a**) Distribution of the occurrence of single-nucleotide polymorphisms (SNPs) variable within chimeras, with a focus on the nature of the substitutions, (**b**) details on differences impacts for coding SNPs, and (**c**) dendrogram based on the dissimilarity of the 40 biological processes gene ontology (GO) terms obtained after term reduction for the genes most impacted by non-silent allelic differences. The relative representation of each GO term is shown as a heatmap.

**Figure 6:**
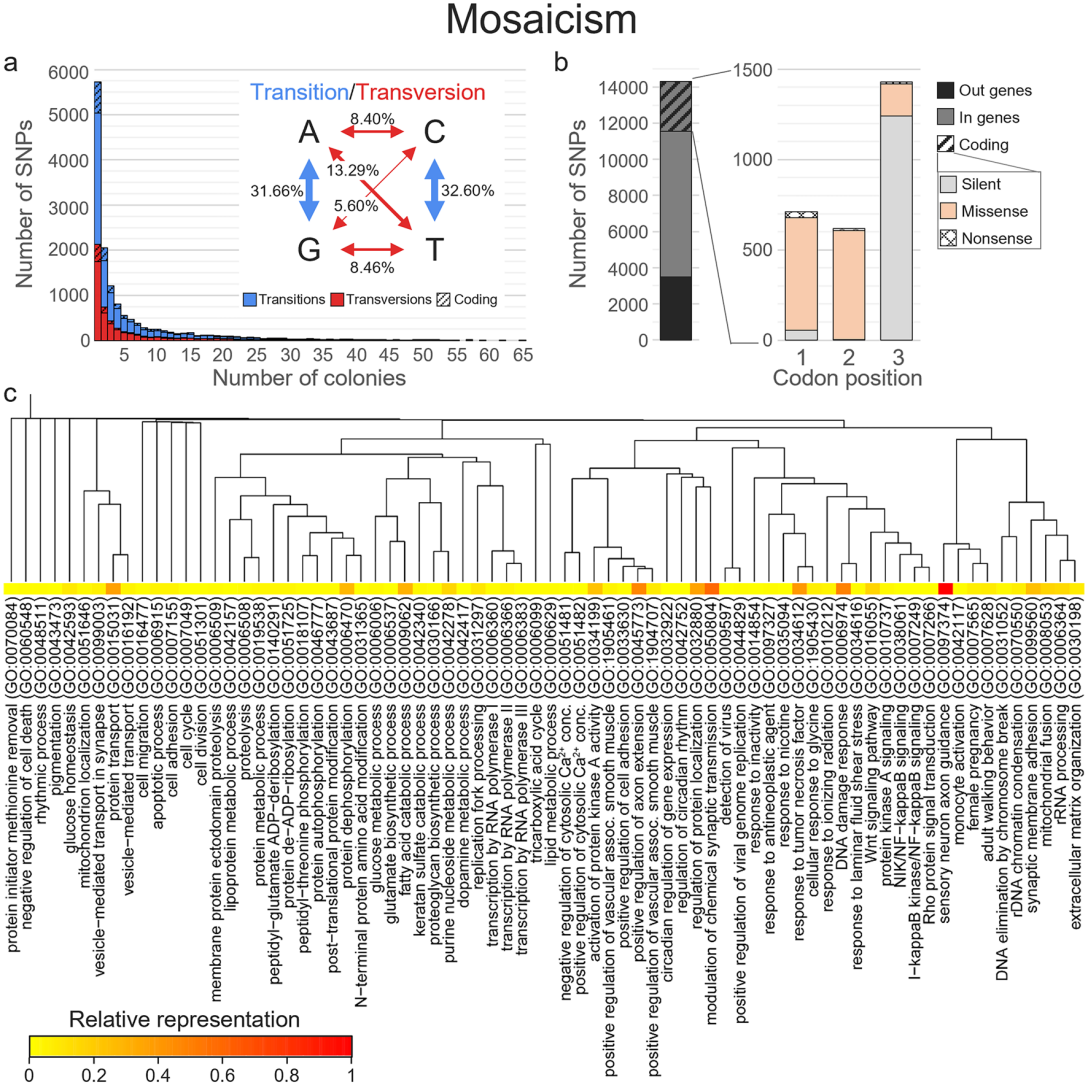
Characterisation of mosaicism. (**a**) Distribution of the occurrence of single-nucleotide polymorphisms (SNPs) variable within colonies, with a focus on the nature of the substitutions, (**b**) details on differences impacts for coding SNPs, and (**c**) dendrogram based on the dissimilarity of the 74 biological processes gene ontology (GO) terms obtained after term reduction for the genes most impacted by non-silent allelic differences. The relative representation of each GO term is shown as a heatmap.

A total of 195,199 allelic differences, corresponding to 38,218 different SNPs (i.e. 64.7% of the filtered SNPs), were found within colonies. More than half of these differences (58.8%), corresponding to 30,799 SNPs, were found in the 21 pairs of chimeric nubbins. Among these differences, 27.1% were found once (i.e. in a single chimera) and 15 (~ 0.05%) differed in all 11 chimeras (Fig. 5a). Most SNPs (89.9%, corresponding to 105,083 differences) were located in 1720 different genes, but only 18.3% (21,482 differences) were coding for 1310 genes (Table S3). More than two thirds of these coding SNPs (70.4%; 15,593 differences) corresponded to the third base of codons, while 17.1% (3454 differences) and 12.5% (2435 differences) corresponded to the first and second bases, respectively (Fig. 5b). Consequently, 70.4% (15,722 differences) of the coding SNPs were silent, 0.6% (107 differences) were nonsense, and the remaining 29.0% (5653 differences) were missense (Fig. 5b; Table S3). Similarly, for mosaicism, 80,529 allelic differences were found in 14,324 SNPs, with 5730 SNPs found once and one found in up to 65 colonies (Fig. 6a). All except 12 differences (< 0.1%) involved genotypes differing by a single allele (i.e. two nubbins were homozygous and the third was heterozygous, or vice versa). Transition/transversion ratio of the differences was 9:5 (Fig. 6a). Three-quarters of the SNPs (75.6%; corresponding to 61,016 differences) were located in 1437 different genes, and 19.3% (16,598 differences) were coding for 620 genes (Table S3). Half of these coding SNPs (51.7%; 8962 differences) corresponded to the third base of codons, while 25.8% (4019 differences) and 22.4% (3617 differences) corresponded to the first and second bases, respectively (Fig. 6b). Thus, 47.1% of the coding SNPs (8052 differences) were silent, 2.0% (279 differences) were nonsense, and the remaining 50.9% (8267 differences) were missense (Fig. 6b; Table S3).

Considering both chimerism and mosaicism, 797 genes were thus impacted by 3126 non-silent SNPs, with up to 59 SNPs and 564 allelic differences on a single gene. More precisely, 252 genes were impacted by both processes, while 396 and 149 were impacted only by chimerism or mosaicism, respectively (Table S3). Thus, for the 648 genes impacted by chimerism, all except 5 genes were previously annotated, and 1869 unique biological processes gene ontology (GO) terms were identified (found in 1 to 28 genes), of which 180 were represented by 50 allelic differences or more (Table S4). These 180 terms were then reduced to 40 by REVIGO (Fig. 5c). Among the most found, terms referring to “ubiquitin-dependent protein catabolic process” (GO:0006511), “cellular response to DNA damage stimulus” (GO:0006974), “positive regulation of transcription by RNA polymerase II” (GO:0045944) or “Wnt signaling pathway” (GO:0016055) were retrieved (Fig. 5c; Table S4). For mosaicism, among the 391 genes impacted and previously annotated, 1387 unique GO terms referring to biological processes were identified (found in 1 to 71 genes; Table S4). Only 332 GO terms had at least 50 allelic differences, which were reduced to 74 terms (Fig. 6c). The most found terms referred to “protein dephosphorylation” (GO:0006470), “peptidyl-tyrosine dephosphorylation” (GO:0035335), “synaptic membrane adhesion” (GO:0099560), “sensory neuron axon guidance” (GO:0097374) or “cellular response to DNA damage stimulus” (GO:0006974; Fig. 6c; Table S4). Interestingly, GO terms referring to processes related to stresses [e.g. “response to tumor necrosis factor” (GO:0034612), “response to ionizing radiation” (GO:0010212)] or to growth [“skeletal system development” (GO:0001501) were also retrieved in the 50 most found terms (Figs. 5c and 6c; Table S4).

## Discussion

Focusing on the study of IGV in *P.* *acuta* populations from Reunion Island using genomic data, our results confirm previous investigations with microsatellites suggesting that IGV is widespread among scleractinians. Indeed, although the detection of mosaic colonies is more complex and extremely sensitive to the defined threshold, and so to the bioinformatics pipeline, 19% of the investigated colonies confidently appeared genetically variable, a proportion that is likely underestimated. Chimeras, on the other hand, were easily and robustly detectable, representing almost 12% of the sampled colonies. Neither process seems correlated with colony surface area, indicating that larger colonies are not necessarily more likely to be mosaics or chimeras. Meanwhile, characterising SNPs that were variable among intra-colonial nubbins, several allelic differences were non-silent and impacted genes with various biological functions. Thus, IGV increases the genetic diversity, genetic plasticity, and adaptive potential of the colonies. This confirms that IGV plays an important role in the maintenance and evolution of scleractinian populations and, more generally, of other organisms.

IGV has been extensively studied in colonial organisms using microsatellites, and its detection relied on the comparison of (multi-locus) genotypes among several nubbins from the same colony^3,11,19,20,25^. Thus, excluding genotyping and scoring errors, any allelic difference reflected a mutation and a genetically variable colony. Accordingly, the probability of detecting a mutation is all the higher as the number of genotyped loci increases (and as the mutation rate is high). But similarly, including additional loci increases the risk of false positives through genotyping and scoring errors^59,60^. Detecting IGV using thousands of high-throughput sequenced loci therefore appears challenging, and it seems obvious that a single allelic difference cannot evidence IGV. Indeed, as an illustration, in this study, the Illumina NovaSeq 6000 platform generated an average (± s.e.) (5.7 ± 0.1) × 10^6^ reads per individual, with a mean phred quality score of 36 (i.e. approximately one error every 4 kbp), resulting in an average 1425 wrong base calls per individual, to which are added DNA replication errors during library preparation. Bioinformatics processing of the reads to reduce these errors is therefore crucial, keeping in mind that mapping^61,62^ and SNP calling^63–65^ can also bring additional genotyping errors.

Testing several SNP calling and filtering parameters to detect IGV, we found that most of them had no noticeable impact on the proportion of IGV colonies. Indeed, these impacts could be compensated by adjusting the genetic dissimilarity threshold defining genetic variability. Only filters based on depth of coverage have a substantial impact, even with the smallest filter tested. This is likely due to the removal of low coverage regions that were not or poorly targeted by our capture protocol or resulted from mapping errors. Additionally, high depths of coverage greatly increase the accuracy of SNP genotyping^63,65^. Consequently, the number of SNPs was severely reduced by those filters, but as was the divergence between sequencing replicates. Overall, this approach also demonstrated the importance of (1) the definition of the genetic dissimilarity threshold on detecting IGV and (2) the inclusion of sequencing replicates. The former needs to be defined specifically for each dataset, as depending on the bioinformatics pipeline, while the latter can help to perceive the usefulness of the different filters. One can also include many sequencing replicates and define the genetic dissimilarity threshold to detect IGV as the mean divergence among those replicates plus some number of times the standard error.

Using the Gaussian mixture model to separate the modes corresponding to genotyping errors and mutations, we estimated a threshold to distinguish colonies presenting IGV or not of 1%. Accordingly, 18 colonies (19%) presented IGV, a number that should be taken cautiously due to its sensitivity to the defined threshold. Using 13 microsatellites, 51% of IGV (47 colonies) was previously detected in this sampling^11^, with 15 colonies detected genetically variable in both the previous and the present studies. Such differences result either from intrinsic differences of the loci (mutation models are different between both types of markers), or from the methodological and technical differences in the definition of IGV with both types of markers (as discussed above). Previous studies investigating the occurrence of IGV in scleractinian populations also found the phenomenon at high rates. For example, in experimental conditions and using nine microsatellite loci, 50% of recently settled juveniles of *Acropora millepora* were found to present IGV^66^. In Lizard Island (northeastern Australia), between 24 and 47% of genetically variable colonies were obtained in five scleractinian taxa: *Acropora florida*, *Acropora hyacinthus*, *Acropora sarmentosa*, *Pocillopora* spp., and *Porites australiensis*, using eight microsatellite loci per taxon^3^. Finally, in Panama, species from the genus *Orbicella* showed up to 38% of genetically variable colonies using 10 microsatellites^25^. According to these previous results, either genomics (based on SNPs harvested from the target-capture of UCEs and exons) underestimates IGV rates (due to conservative filters to exclude genotyping errors) or microsatellites overestimate them (e.g., due to non-excluded genotyping errors). In any case, results from this study are hardly comparable to previous ones, and no other study has yet evaluated the proportion of IGV in natural coral populations using genomics to our knowledge. Future studies should adopt a comparative framework with multiple coral species exhibiting different life history traits to better understand how mosaicism and chimerism vary among species and may be favoured by certain traits. Are branching corals more favourable due to their generally higher growth rate or because branch tissues are in less contact with the rest of the colony? How do reproductive strategies, in particular asexual reproduction, influence IGV? The discovery of this phenomenon remains recent, particularly in scleractinians, and several questions still need to be addressed.

Unlike the threshold to distinguish colonies presenting IGV or not, the one distinguishing mosaicism and chimerism was unambiguous, with a large gap separating both mechanisms in the distribution of the percentages of different alleles. Accordingly, 11 chimeras (12% of the colonies) were detected, a slightly higher number than previously found using microsatellites (nine in Oury et al.^11^). In particular, six chimeras were detected by both types of markers (corresponding to chimeras C3 to C7 and C9 in Oury et al.^11^). The three that were not retrieved using genomics were those with the fewest number of different alleles within the genotyped microsatellite loci, suggesting a previously defined threshold potentially too low. Conversely, five additional chimeras were detected using genomics, all previously considered mosaics, suggesting that the microsatellite markers used were not enough polymorphic to distinguish some chimeras. Although consistent with previous investigation based on microsatellites, the proportion of chimeric colonies appear high compared to Schweinsberg et al.^3^ findings (from 2.4 to 4.5% of chimeras for three *Acropora* species, *Pocillopora* spp., and *Porites australiensis*), but low compared to Puill-Stephan et al.^66^ ones (50% in *A.* *millepora*). However, the latter study investigated chimerism among recruits, where the proportion is higher and progressively reduced due to the death of one or all of the genotypes involved^66^.

Chimerism has been reported rarer than mosaicism^3,67,68^ due to their respective mechanisms of formation. This was not the case in this study (7% and 12% of the colonies were mosaic and chimeric, respectively), but it seems to be a direct consequence of the underestimation of the proportion of IGV colonies. Indeed, while chimeras were easily detected, mosaics remained in the ambiguous zone between true mutations and genotyping errors and were thus possibly poorly detected.

Noteworthy, we found no obvious relationship between colony surface area, as a proxy of colony age and of distance among intra-colonial nubbins, and the mean intra-colonial proportion of different alleles. Mutations should accumulate over time, therefore bigger (and older assuming that growth rate is relatively constant) colonies, from which we collected more distant nubbins, should be more variable. Some mechanisms might exist to correct mutations along the colony, however this hypothesis must be taken cautiously since it was not the aim of this study. Colony parts may also die and be recolonised, hence influencing the genotypes spatio–temporal distribution. The latter distribution has previously been investigated in chimeric colonies of the coral *Stylophora pistillata* using eight microsatellites, revealing intermixed and disproportionate genotype distributions^26^. Further studies mapping the extent of mutations in colonies using genomics, as already done in plants^69^, could help understanding how mutations accumulate with growth.

Genomics also allows for identification and characterisation of intra-colonial allelic differences. Over all differences investigated, few (19.5%) were coding and fewer (7.3%) were non-silent. On one hand, this suggests that although widespread, IGV poorly impacts genes, but on the other hand, these few impacts still increase genetic diversity and plasticity, synonymous with an increase in the adaptive potential^70^. Besides, chimerism was responsible for more than half of the allelic differences found, whereas investigated in only 21 pairs of intra-colonial nubbins (7.7%). This demonstrates that chimerism strongly and rapidly increases the genetic variability and adaptive potential of colonies. Chimeras of the coral *Stylophora pistillata* have also been shown to express stress-responsive genes at higher levels, which may increase their robustness to face environmental stresses^71^. Most of the biological functions we found impacted by chimerism involved regulatory mechanisms, which may induce a similar effect on colonies’ resistance to stresses. Transcriptomics would allow to quantify the expression of these genes, but also to confirm an increase of plasticity.

Although chimeras are genetically more diverse, they do not have new alleles, and chimerism is not a source of genetic innovation in the population. Indeed, the different alleles were inherited parentally and if the individuals had not fused, they would still have been found in the population (assuming parents and/or offspring survive). Conversely, mosaicism, through mutations, represents a source of new alleles. Even if these mutations concern somatic cells, they can be propagated with clonal propagation processes (e.g. fragmentation or budding).Some mutations might be detrimental (e.g. the nonsense ones), but as > 99% were found on a single allele for non-chimeric colonies, the initial allelic state is still represented, and gene functions are thus still maintained. Some non-silent mutations might haphazardly induce beneficial genetic modifications that could be positively selected under ongoing global changes. Unfortunately, we were unable to detect positively selected SNPs using outlier detection approaches^72^ with our dataset (analyses not shown), but the diversity of the biological processes associated with the impacted genes demonstrates a large panel of potentially impacted functions, some of which are directly related to responses to environmental stresses. Using a seascape genomics approach in the corals *Acropora millepora*, *P.* *acuta*, and *P.* *damicornis* from New Caledonia, a previous study^73^ identified SNPs correlated with heat stress gradients and were located in proximity of genes involved in cellular responses against heat. This suggests heat stress adaptations, but also confirms positively selected SNPs under heat stress. Thenceforth, IGV, both through mosaicism and chimerism, represents a potential lifeline and a source of genetic innovation and genetic diversity for scleractinians.

These results should nevertheless be taken cautiously as target-sequenced regions were UCEs and exon loci, i.e. little variable and biased towards coding regions. These conserved regions are nevertheless flanked by introns and other more variable regions that are also sequenced, making them suitable for phylogenomic^38,74,75^ to population genomic^39,76^ studies. Other high-throughput methods sequencing random regions (e.g. RADseq) or the whole genome might be more appropriate for characterising the potential roles of IGV, allowing us to estimate less biased proportions of coding vs non-coding SNPs. However, the accuracy of distinguishing between colonies variable or not, and between mosaics and chimeras, still relies on the accurate definition of the thresholds. Finally, only biallelic SNPs were considered in this study for analytical reasons, thus missing a high proportion of intra-colonial genetic differences (e.g. tri- and tetra-allelic SNPs, insertions, and deletions) and potentially underestimating IGV and its role. Nonetheless these results allowed a first investigation through the potential roles of IGV at the genetic level. They confirmed the genetic dimension of the advantages provided by IGV and the importance of this phenomenon in the maintenance and evolution of scleractinian populations.

In conclusion, although making the detection of mosaic colonies more complex, genomics represents a valuable tool for investigating the potential roles of IGV from a genetic point of view. Our results confirm, on one hand, the presence of IGV in high proportions in *P.* *acuta* populations from Reunion Island, and in scleractinian populations in general, and on the other hand, provide new insights into the roles of IGV. They also demonstrate how important the definition of the threshold to distinguish colonies presenting IGV or not is, and how it is dependent on the bioinformatics pipeline used and its chosen parameters. IGV, therefore, appears as a source of genetic diversity and genetic plasticity for organisms, and it seems undeniable that it will have a role to play in the future of coral reefs.

### Supplementary Information

Supplementary Information.

Supplementary Tables.

## Data Availability

All data underlying this article is available online. Raw sequencing reads were deposited on NCBI (BioProject PRJNA836440). Datasets, example code, and scripts are available at http://doi.org/10.5281/zenodo.10633675.
