# Supplementary material for: Investigating the potential roles of intra-colonial genetic variability in Pocillopora corals using genomics
Source: Sci Rep. 2024 Mar 18;14:6437. doi: 10.1038/s41598-024-57136-5 (PMC10948807; doi:10.1038/s41598-024-57136-5)

Supplementary Information for

Investigating the potential roles of intra-colonial genetic variability in *Pocillopora* corals using genomics

Nicolas Oury* & Hélène Magalon

* email: nicolasoury@hotmail.fr

Content:

Table S1. Softwares, tools, and parameters used for datasets construction 2

Table S2. Identification datasets and single-nucleotide polymorphism (SNP) filtering steps 3

Table S3. Numbers of SNPs and differences variable within colonies for each gene of the *Pocillopora verrucosa* genome .... see excel file

Table S4. Biological processes gene ontology (GO) terms impacted by intra-colonial genetic variability .... see excel file

Fig. S1. Distributions of the pairwise percentages of different alleles among the 282 nubbins.......... 4

Fig. S2. Dendrogram of the 282 nubbins based on pairwise percentages of different alleles 5

Fig. S3. Species identification of the nubbins 6

Fig. S4. Maximal intra-colonial percentage of different alleles between nubbins for each colony and each dataset 7

Fig. S5. Correlation between colony surface and intra-colonial percentage of different alleles 8

Table S1. Softwares, tools, and parameters used for datasets construction.

Function	Tool	Software/package	Parameters	Ref.
**Preliminary bioinformatics steps**				
Quality control	FastQC v0.11.7<br>MultiQC v1.7		N/A<br>N/A	[1]<br>[2]
Adapter trimming and low-quality bases removal	Trim Galore! v0.6.0		--paired R1.fq R2.fq<br>--trim-n<br>--illumina<br>--nextseq 20	[3]
**Species identification of the nubbins**				
Reads mapping	BWA v0.7.17		mem reference.fa R1.fq R2.fq	[4]
Reads sorting	SortSam	Picard v2.20.7	SORT_ORDER=coordinate	[5]
Duplicates marking	MarkDuplicates	Picard v2.20.7	ASO=coordinate	[5]
Local realignment	RealignerTargetCreator<br>IndelRealigner	GATK v3.8.1	-R reference.fa<br>-R reference.fa	[6]
SNP genotyping	mpileup<br>call<br>filter	BCFtools v1.9	-A -B -I -a AD,DP,SP,INFO/AD<br>-R list.SNPs -f reference.fa<br>-m<br>-S ‘.’ -e ‘FORMAT/DP<12’<br>-S ‘.’ -e ‘FORMAT/SP>13’	[7]
**Intra-colonial genetic variability analysis**				
Reads mapping	BWA v0.7.17		mem reference.fa R1.fq R2.fq	[4]
Reads sorting	SortSam	Picard v2.20.7	SORT_ORDER=coordinate	[5]
Duplicates marking	MarkDuplicates	Picard v2.20.7	ASO=coordinate	[5]
Local realignment	RealignerTargetCreator<br>IndelRealigner	GATK v3.8.1	-R reference.fa<br>-R reference.fa	[6]
SNP calling and filtering	mpileup<br>call<br>filter	BCFtools v1.9	-A -I -a AD,DP,SP,INFO/AD<br>**-Q XX -q XX** -f reference.fa<br>-mv<br>-i ‘**QUAL>=XX**’<br>-S ‘.’ -e ‘FORMAT/**DP<XX**’<br>-S ‘.’ -e ‘FORMAT/**SP>13**’<br>-m2 -M2<br>- **AN>XX**	[7]

References

[1] http://www.bioinformatics.babraham.ac.uk/projects/fastqc/

[2] Ewels P, Magnusson M, Lundin S, Käller M (2016) MultiQC: summarize analysis results for multiple tools and samples in a single report. Bioinformatics 32:3047–3048. doi: 10.1093/bioinformatics/btw354

[3] http://www.bioinformatics.babraham.ac.uk/projects/trim_galore/

[4] Li H, Durbin R (2009) Fast and accurate short read alignment with Burrows-Wheeler transform. Bioinformatics 25:1754–1760. doi: 10.1093/bioinformatics/btp324

[5] https://broadinstitute.github.io/picard/

[6] McKenna A, Hanna M, Banks E, Sivachenko A, Cibulskis K, Kernytsky A, Garimella K, Altshuler D, Gabriel S, Daly M (2010) The Genome Analysis Toolkit: a MapReduce framework for analyzing next-generation DNA sequencing data. Genome Res 20:1297–1303. doi: 10.1101/gr.107524.110

[7] http://samtools.github.io/bcftools/

Table S2. Identification datasets and single-nucleotide polymorphism (SNP) filtering steps. Final datasets used for analyses are highlighted in grey.
N_{ind}, N_{loci}, N_{SNP}, and N_{GT}: numbers of individuals, loci, SNPs, and genotypes, respectively, *%NA* and *%NA*$_{ind}$: percentages of missing data for the overall dataset and per individual, respectively, *Δrep*: mean divergence between sequencing replicates of the same nubbin, DP: genotype depth of coverage and SP: strand bias.

**Filters**	N_{ind}	N_{loci}	N_{SNP}	N_{GT}	***%NA***	***Δrep***
**Oury et al. (2023a) identification dataset**						
Initial genotype calling	285	1 559	1 559	440 520	0.85%	0.30%
& DP ≥ 12	285	1 559	1 559	426 548	4.00%	0.27%
& SP < 13	285	1 559	1 559	423 622	4.66%	0.22%
& *%NA*$_{ind}$ < 35%	282	1 559	1 559	423 619	3.64%	0.22%
& no replicate	276	1 559	1 559	414 603	3.64%	-
& no clone	15	1 559	1 559	22 724	2.83%	-
+ Oury et al. (2023a) dataset	376	1 559	1 559	551 579	5.90%	-
**Oury et al. (2023b) identification dataset**						
Initial genotype calling	285	1 493	1 493	422 266	0.76%	0.55%
& DP ≥ 12	285	1 493	1 493	412 066	3.16%	0.44%
& SP < 13	285	1 493	1 493	407 623	4.20%	0.36%
& *%NA*$_{ind}$ < 35%	282	1 493	1 493	407 618	3.18%	0.36%
& no replicate	276	1 493	1 493	398 940	3.19%	-
& no clone	15	1 493	1 493	21 787	2.71%	-
+ Oury et al. (2023b) dataset	110	1 493	1 493	159 391	2.95%	-

References

Oury N, Noël C, Mona S, Aurelle D, Magalon H (2023a) From genomics to integrative species delimitation? The case study of the Indo-Pacific *Pocillopora* corals. Mol Phylogenet Evol 184:107803. doi: 10.1016/j.ympev.2023.107803

Oury N, Mona S, Magalon H (2023b) Same places, same stories? Genomics reveals similar structuring and demographic patterns for four *Pocillopora* coral species in the southwestern Indian Ocean. J Biogeogr n/a:1–15. doi: 10.1111/jbi.14788

Figure S1. Distributions of the pairwise percentages of different alleles among the 282 nubbins for (a) the 1,559 SNPs from Oury et al. (2023a) and (b) the 1,493 SNPs from Oury et al. (2023b). Red dashed lines indicate mean (over nine comparisons) pairwise distances among sequencing replicates of the same nubbin. Grey polygons indicate gap zones.

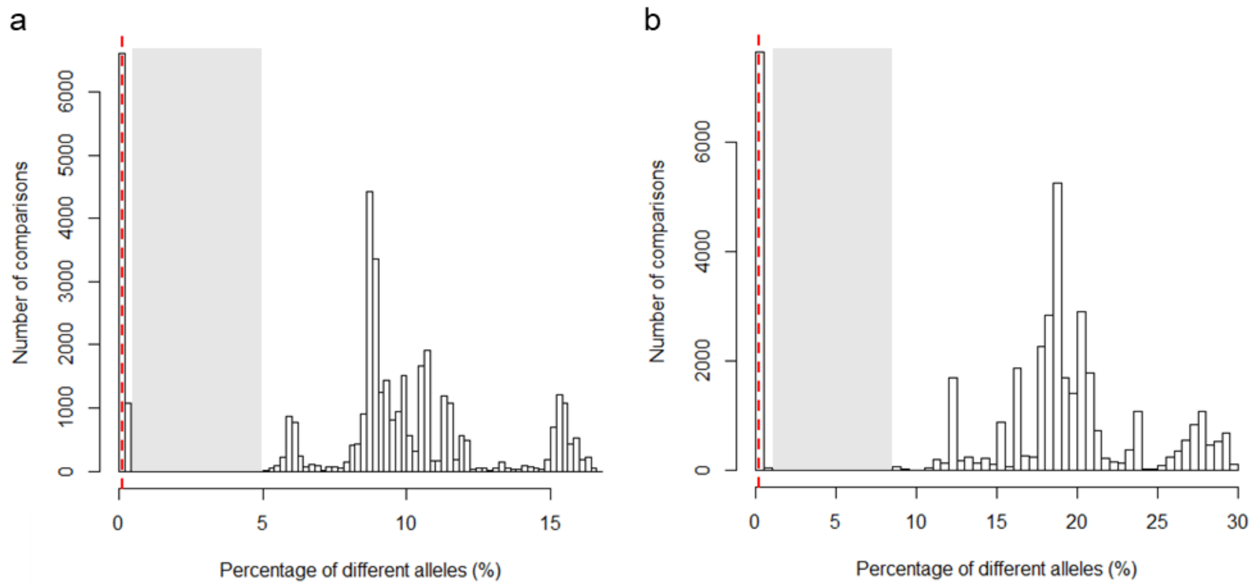

References

Oury N, Noël C, Mona S, Aurelle D, Magalon H (2023a) From genomics to integrative species delimitation? The case study of the Indo-Pacific *Pocillopora* corals. Mol Phylogenet Evol 184:107803. doi: 10.1016/j.ympev.2023.107803

Oury N, Mona S, Magalon H (2023b) Same places, same stories? Genomics reveals similar structuring and demographic patterns for four *Pocillopora* coral species in the southwestern Indian Ocean. J Biogeogr n/a:1–15. doi: 10.1111/jbi.14788

Figure S2. Dendrogram of the 282 nubbins based on pairwise percentages of different alleles for the 1,559 SNPs from Oury et al. (2023a). Branches were coloured according to the 15 clonal lineages and coloured tip labels denote chimeras (one colour per colony).

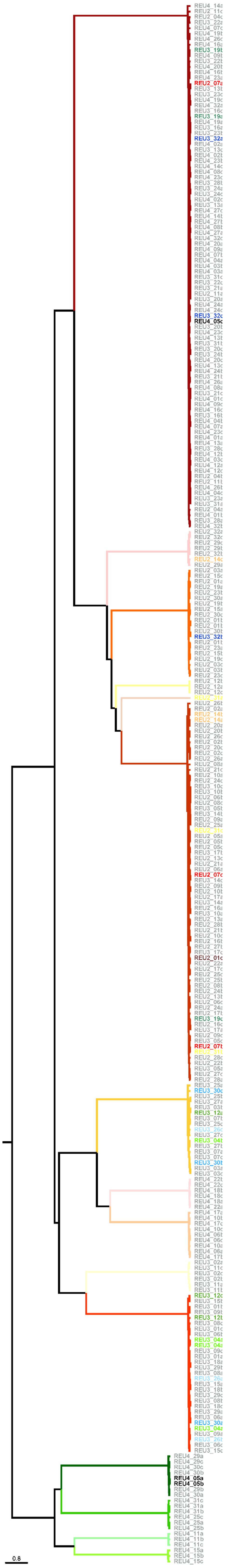

Reference

Oury N, Noël C, Mona S, Aurelle D, Magalon H (2023a) From genomics to integrative species delimitation? The case study of the Indo-Pacific *Pocillopora* corals. Mol Phylogenet Evol 184:107803. doi: 10.1016/j.ympev.2023.107803

Figure S3. Species identification of the nubbins. (a) sNMF assignments from $K = 2$ to $K = 10$ for the 15 clonal lineages combined with the species delimitation dataset of Oury et al. (2023a; 361 colonies × 1,559 SNPs) and (b) sNMF assignments at $K = 3$ for the 15 clonal lineages combined with the *Pocillopora acuta* truncated dataset of Oury et al. (2023b; 95 colonies × 1,493 SNPs).

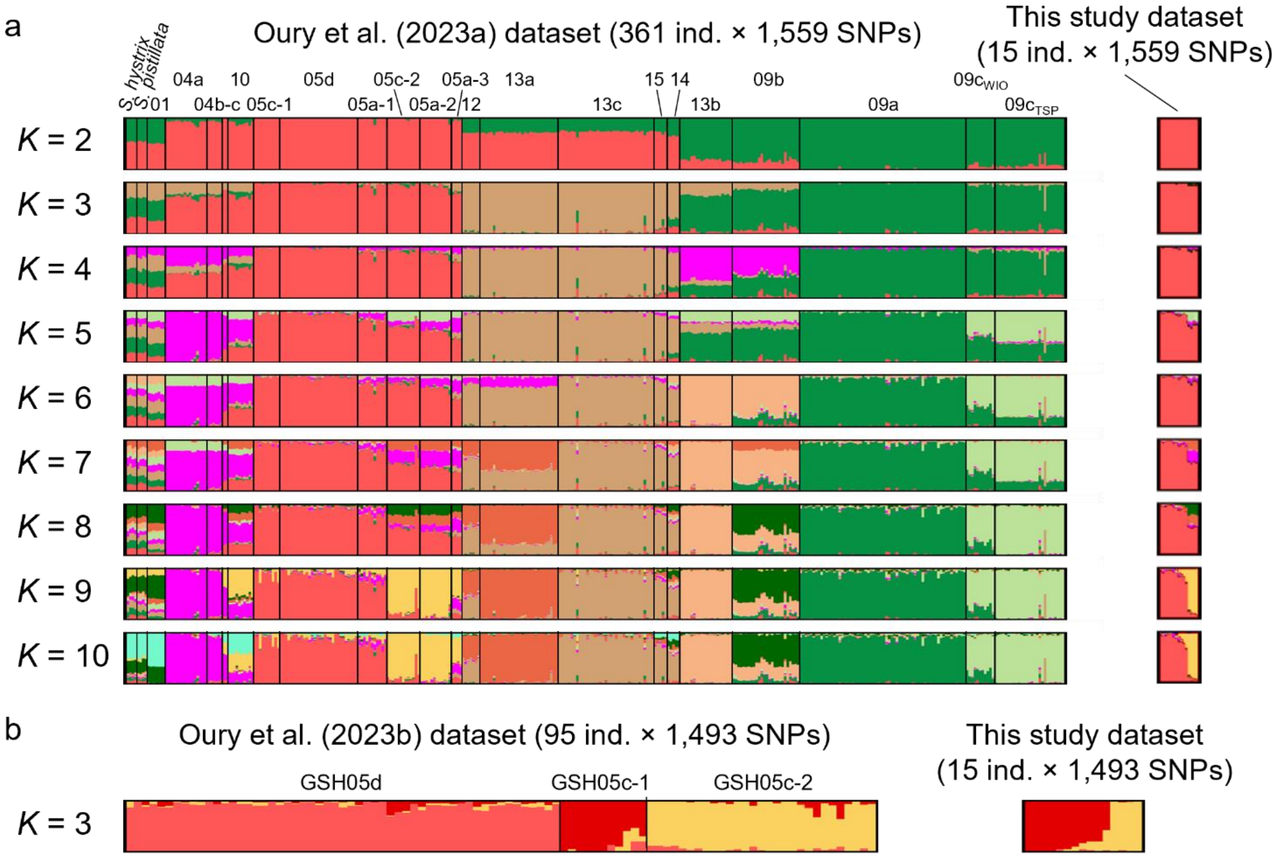

References

Oury N, Noël C, Mona S, Aurelle D, Magalon H (2023a) From genomics to integrative species delimitation? The case study of the Indo-Pacific *Pocillopora* corals. Mol Phylogenet Evol 184:107803. doi: 10.1016/j.ympev.2023.107803

Oury N, Mona S, Magalon H (2023b) Same places, same stories? Genomics reveals similar structuring and demographic patterns for four *Pocillopora* coral species in the southwestern Indian Ocean. J Biogeogr n/a:1–15. doi: 10.1111/jbi.14788

Figure S4. Maximal intra-colonial percentage of different alleles between nubbins for each colony and each dataset (indicated above; see Table 1). Red dots show the 11 colonies with higher maximal intra-colonial distances.

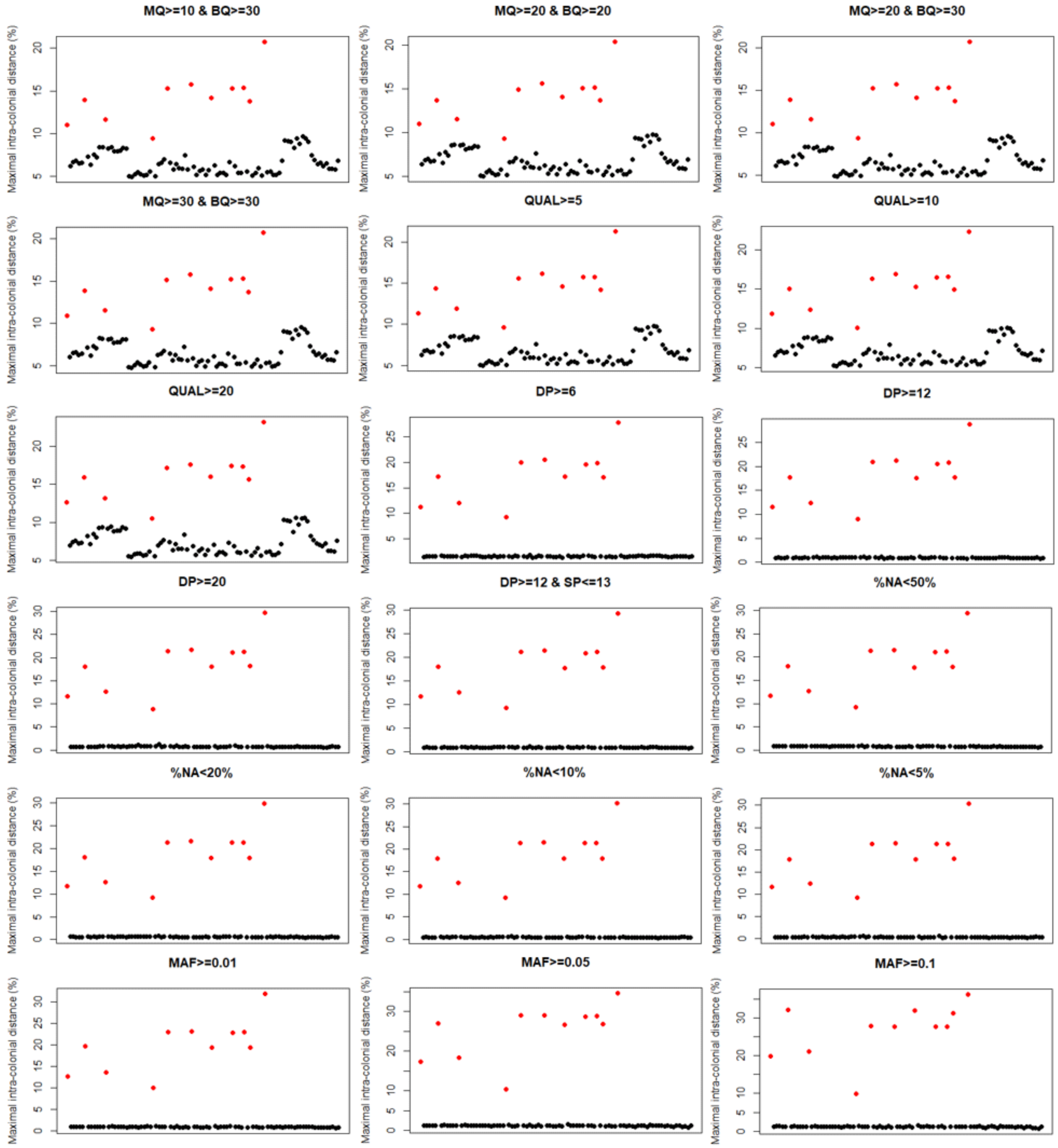

Figure S5. Correlation between colony surface and mean intra-colonial percentage of different alleles, considering only chimeric (a) or mosaic (b) nubbins.

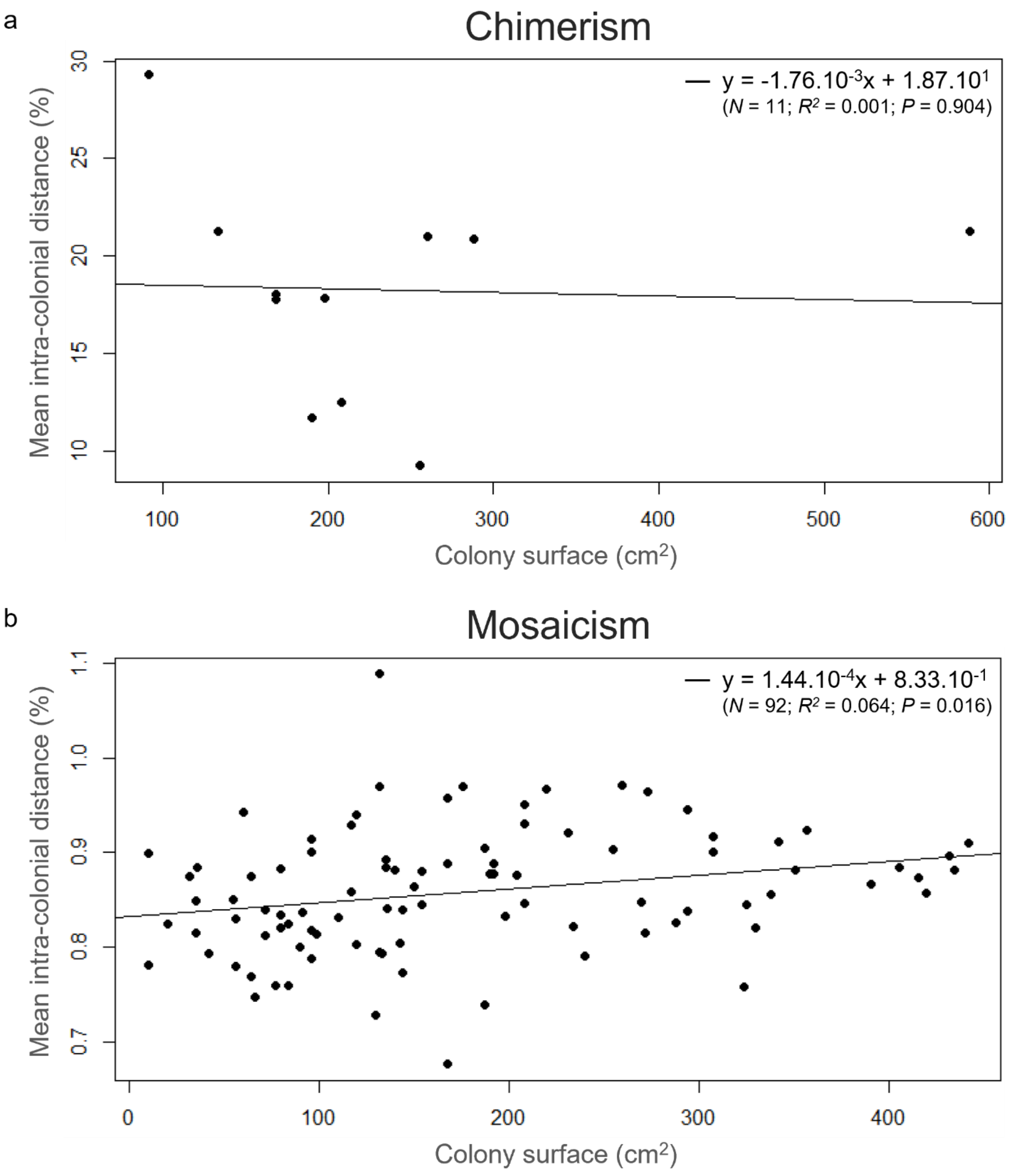

Supplement: Supplementary file 1 — Supplementary Information. [file 41598_2024_57136_MOESM1_ESM.pdf]
